# Time-consciousness in computational phenomenology: a temporal analysis of active inference

**DOI:** 10.1093/nc/niad004

**Published:** 2023-03-17

**Authors:** Juan Diego Bogotá, Zakaria Djebbara

**Affiliations:** Department of Social and Political Sciences, Philosophy, and Anthropology, University of Exeter, Exeter, United Kingdom; Grupo de investigación Filosofía y cognición, Universidad Nacional de Colombia, Bogotá, Colombia; Department of Architecture, Design, Media, and Technology, Aalborg University, Rendsburggade 14, Aalborg 9000, Denmark; Biological Psychology and Neuroergonomics, Technical University of Berlin, Fasanenstraße 1, Berlin 10623, Germany

**Keywords:** active inference, temporality, temporal analysis, computational phenomenology, consciousness

## Abstract

Time plays a significant role in science and everyday life. Despite being experienced as a continuous flow, computational models of consciousness are typically restricted to a sequential temporal structure. This difference poses a serious challenge for computational phenomenology—a novel field combining phenomenology and computational modelling. By analysing the temporal structure of the active inference framework, we show that an integrated continuity of time can be achieved by merging Husserlian temporality with a sequential order of time. We also show that a Markov blanket of the present moment integrates past and future moments of both subjective temporality and objective time in an asynchronous manner. By applying the integrated continuity, it is clear that active inference makes use of both subjective temporality and objective time in an integrated fashion. We conclude that active inference, on a temporal note, qualifies as a computational model for phenomenological investigations.

## Introduction

Human experience is characterized by its continuous temporal structure. Any moment of our experience appears as a coherent flow that goes from the immediate past to the immediately incoming moment. It has been argued that this temporal continuity must be accounted for because of its ubiquity (see e.g. Gibson 1986; Husserl 1991, 2001b; Van Gelder 1999; Bergson 2004, Yoshimi 2016; Buonomano 2017). Indeed, it is impossible to think about experience without presupposing its continuous temporal flow.

To address the temporal flow of experience, various computational modelling frameworks often make use of static computations restricted to discrete jumps in time determined by either the data or the system in use (see e.g. Elman 1990; Grush 2005; Wiese 2017). Within these approaches, however, the subjective, dynamic continuum appears not to correspond to the objective, discrete time point that computer models employ (Jazayeri and Shadlen 2010; Shi *et al.* 2013; Fischer and Whitney 2014; Glasauer and Shi 2022). Whereas we subjectively experience a flowing ‘specious present’ (James 1950; Varela 1999; Husserl 2001b) in which the distinction between past, present, and future blurs; objectively we conceive of time as a rigid ordering of events. These differences lead to a challenge for computational models of experience: how can such models overcome the dissonance between the ‘continuous’ and ‘specious’ temporal structure in human experience (call it ‘subjective temporality’) and the ‘discrete’ and ‘rigid’ temporal structure (call it ‘objective time’) of such computational models?

By focusing on a particular modelling technique, active inference (Friston *et al.* 2017), we ask specifically whether there is a confluence between how subjective temporality and objective time are expressed within the model. More specifically, how can active inference models account for the flowing and continuous temporal structure in human experience, given their static and discrete jumps in time? In sum, this paper addresses the compatibility between the temporal nature of human experience and the temporal structure in the framework of active inference. In particular, here, we formalize some core aspects of the Husserlian approach to subjective temporality (which emphasizes the continuity of time) in the language of Bayesian networks (which typically operate in a sequential fashion) to properly assess the temporal structure of the active inference framework.

Given the importance of the temporal structure of experience within both phenomenology and cognitive science, this paper is a crucial step towards computational phenomenology—a novel naturalized phenomenology approach that focuses on the idea of using generative modelling techniques to model the dynamics of consciousness. For phenomenologists, temporality is an essential and basic structure of experience. For instance, Husserl (2001a, pp. 170–171) claims that that the structure of time-consciousness is ‘a universal, formal framework, in a synthetically constituted form in which all other possible syntheses must participate’, and therefore temporality can be interpreted as the ‘A’ in the ABCs of consciousness and subjectivity. Correlatively, the naturalization of the Husserlian approach to the temporal structure of experience can be seen as a ‘benchmark’ for modelling approaches to consciousness within cognitive science, given how several of such approaches try to explicitly account for it (e.g. Van Gelder 1999; Varela 1999; Grush 2006; Wiese 2017). Therefore, our proposal tackles the basic structure of experience to show the potential of active inference and, more specifically, computational phenomenology while, simultaneously, showing how computational models can account for the continuous temporal structure of experience.

In what follows, we first (in the section ‘Computational Phenomenology and the Project of Naturalization’) frame our proposal within the projects that try to naturalize Husserlian phenomenology. More specifically, we conceive of our view as a crucial step towards computational phenomenology (Ramstead *et al.* 2022). In line with other naturalized phenomenology approaches, we see Husserl’s analyses of time-consciousness as an ‘acid test’ for any of such approaches (see Varela 1999). That is why, in the section ‘Husserlian time-consciousness’, we present some main points of Husserl’s analyses and identify two phenomenological criteria that computational models of subjective temporality must comply with. Then, in the section ‘Objective Time: Thermal Time’, we characterize objective time under the lens of Carlo Rovelli’s (1993) ‘thermal time hypothesis’, emphasizing the difference between subjective temporality and objective time. The section ‘An Active Inference Model of Temporality’ represents the core of our proposal. In this section, we analyse the temporal structure of active inference, showing that an interpenetrated and sequential temporal structure can be integrated into an ‘integrated continuity’. In doing so, we reconsider the application of Markov blankets as the boundary of ‘things’ and instead apply it to the temporal structure to identify the system that describes the present moment under active inference without violating the Markov property (Section 4.3.). This analysis is then evaluated against the phenomenological criteria identified earlier to examine whether a confluence between how subjective temporality and objective time is expressed in active inference. We show that the temporal structure that is disclosed via phenomenological analysis is directly compatible with the temporal structure suggested in the active inference framework. Lastly, in the section ‘An Active Inference Model of Temporality’, we briefly mention some possible applications based on our analysis.

## Computational phenomenology and the project of naturalization

During the last few decades, there have been several proposals for the ‘naturalization’ of Husserlian phenomenology (e.g. Petitot *et al.* 1999; Gallagher 2003; Thompson 2007; Yoshimi 2016). The guiding thread common to these proposals is that the methods, concepts, and descriptions used within the phenomenological tradition inaugurated by Edmund Husserl may be useful for scientific attempts to explain consciousness. However, if phenomenology is to be linked with empirical research, the former must be ‘naturalized’ (i.e. made continuous with the natural sciences) given its transcendental considerations (which are often contrasted with naturalistic claims in empirical science). Put simply, transcendental phenomenology refers to the philosophical study of the constitutive dimension of subjectivity. From this perspective, consciousness is understood as the condition for the meaningfulness of the world and worldly entities that are studied by empirical science. For a review of different senses of naturalization in the context of naturalized phenomenology, see Ramstead (2015). For critical analyses of the idea of a naturalized phenomenology, see Zahavi (2004, 2010, 2013).

We consider that a formal modelling framework like active inference may serve as a link between phenomenology and empirical science. Our view is not that phenomenological descriptions and analyses are to be reduced to mathematical treatment, assuming that consciousness is a natural process that can be addressed by the natural sciences if it is put into a proper mathematical language (Petitot 1999a). Rather, from our perspective, the connection between phenomenology and mathematical models is not of reduction of the former into the latter but of ‘phenomenologization’ in which the models are made meaningful by giving methodological priority to phenomenology.

A critical analysis of the possibility of the phenomenologization of natural science is beyond the scope of this paper. Suffice it to say that the idea of the phenomenologization co-emerged with that of the naturalization of phenomenology. As Zahavi (2004, p. 344) recounts, during a meeting in Paris in 2000, Francisco Varela replied to a question posed by him claiming that a second complementary volume to the often-cited ‘Naturalizing Phenomenology’ (Petitot *et al.* 1999b), titled ‘Phenomenologizing Natural Science’, was originally planned. That second volume was never published. Ever since, however, the thought that a proper naturalization of phenomenology requires a phenomenologization of natural science has been explored by some philosophers (see e.g. Zahavi 2011; Thompson 2011, pp. 219–2020; Vörös 2014; Gallagher 2018). All these approaches have in common the call for a non-reductive and non-objectivist science.

We believe that, in the context of consciousness studies, a step towards phenomenologization can be done by approaching the structures of consciousness via modelling frameworks. More specifically, we consider that models of consciousness should be phenomenologized by first acknowledging that the variables of such models only make sense in reference to the structures of consciousness that are revealed via careful phenomenological descriptions and analyses. In other words, a given model of consciousness requires a prior phenomenological investigation on the structures of consciousness that the scientist is interested in. Only after acquiring the relevant phenomenological evidence, an appropriate model can be used to represent it. Importantly, a properly phenomenologized model can, in turn, constrain our understanding of the underlying cognitive mechanisms (Ramstead *et al.* 2022, p. 17).

This paper represents the first step in the direction just outlined. We aim to show that the temporal structure of consciousness as analysed within Husserlian phenomenology can be properly modelled using active inference. From this perspective, our view can be framed within the ‘computational phenomenology’ project proposed by Ramstead *et al.* (2022). Computational phenomenology is a novel perspective on the formalization and modelling of the descriptions and analyses of ‘lived experience’ in philosophical phenomenology (i.e. the work of Husserl, Merleau-Ponty, etc.) employing generative modelling approaches such as active inference. The formalization of such descriptions and analyses can be seen as an important component of the broader project of the naturalization of phenomenology in the sense just outlined.

## Husserlian time-consciousness

In line with some cognitive scientists, we believe that the naturalization of Husserl’s analyses on time-consciousness functions as an ‘acid test’ for any naturalized phenomenology (Varela 1999, p. 267; see also Van Gelder 1999; Grush 2006; Wiese 2017). The main reason behind this thought is the importance of the structure of subjective temporality for any account of consciousness. Within the phenomenological tradition, subjective temporality enjoys a privileged position within the structures of consciousness. As Zahavi (2003, pp. 86–87) points out, there are two main reasons why the analysis of temporality is a central aspect of Husserlian phenomenology. First, without an understanding of subjective temporality, the analysis of intentionality would remain incomplete. Objects are experienced as identities within a manifold of appearances. Such identities are fundamentally temporal. Second, consciousness itself is experienced as a temporal flow of experiences. Therefore, a phenomenological analysis of temporality would disclose the structure of both experienced objects and subjectivity itself. From this perspective, temporality is so important that its phenomenological structure is presented as presupposed by any other constitutive process of consciousness (Husserl 2001a, pp. 170–171). This is the reason why Husserl claimed that the analyses on temporality are ‘[…] perhaps the most important in the whole of phenomenology’ (Husserl 1991, p. 346).

In this section, we introduce some of the main points of the Husserlian analyses of time-consciousness to identify two criteria that any computational model of subjective temporality must comply with. It must be acknowledged that, throughout his whole career, Husserl attempted to make sense of the intrinsic temporality of experience (see Husserl 1991, 2001a, 2006), and therefore, it would be misleading to claim that there is a single Husserlian view on time-consciousness. An exhaustive exegetical work on Husserl’s phenomenology of subjective temporality is beyond the scope of this paper. Instead, here we focus on some of the elements that remain relatively unchanged throughout Husserl’s analyses.

**Figure 1.**
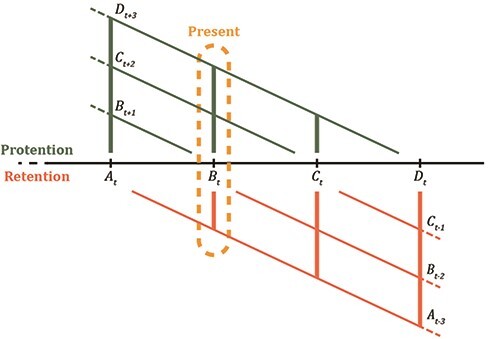
A schematic of the Husserlian analysis of subjective temporality (inspired by Husserl 2001b, p. 22). The horizontal x-axis refers to the objective time, while the axes above the x-axis refer to the protention and the axes below refer to retention in subjective time.

To illustrate the temporal structure of experience, consider a melody that goes from A to B, to C, to D (Fig. 1). When listening to the melody, each tone appears as distinct from one another. However, the tones are connected in experience in such a way that they constitute a larger whole—the perceived melody. It is not simply that C comes after B and A. Rather, C is experienced as arising from B, which itself was experienced as arising from A. The experiential meaning of C is constituted by its coming after B and A. It is as if A and B were still experienced when hearing C—but not experienced as happening right now, but rather as ‘just-past’. Moreover, when hearing C, there is something about it that suggests that something else is about to come (i.e. note D). Imagine that instead of D, there is an abrupt end to the melody just after C. Such an abrupt end would be surprising, which suggests that when hearing C there is an anticipation of something else apart from an abrupt end. Therefore, at any given moment, not only our experience is directed towards what is currently happening, but it also intends the ‘just-past’ and the ‘about-to-occur’. The intending of the ‘just-past’ is what Husserl (2001b) calls ‘retention’, whereas he uses the notion ‘protention’ to refer to the intending of the ‘about-to-occur’. Additionally, the intending of the ‘now-phase’ of experience (i.e. what is happening right now, in between what is retained and what is protended) is referred to as a ‘primal impression’ (Husserl 1991, 2001a). Importantly, retention, primal impression, and protention occur simultaneously, which means that at any given moment, we are simultaneously experiencing the ‘just-past’, the ‘now-phase’, and the ‘about-to-occur’ of experience. Therefore, the present is experienced as a ‘duration-block’, as James (1950, p. 574) would describe it. This duration-block present is referred to by Husserl as the ‘living present’ (e.g. Husserl 1991, p. 56)—a somewhat rigid structure of retentions, primal impressions, and protentions—that is nevertheless continuously flowing.

Husserl’s views on time-consciousness are consistent with process philosophy. Process philosophers argue that being is dynamic and so the dynamic nature of being should form the core of any analysis of reality and our place within it (Rescher 2000; Dupré 2014; Seibt 2022). From a processual perspective, studying human experience is not a study of the properties of a stable object but the processes that enable the system to maintain itself sufficiently for it to continue to function (Bergson 2001, 2007; Dupré 2012, 2014; Nicholson and Dupré 2018). It is by linking Husserl’s views to process philosophy that it becomes especially salient how, within the Husserlian framework, subjective temporality is essentially continuous. To appreciate how Husserl’s approach fits within process philosophy, it is useful to consider the connections between his phenomenology of inner time-consciousness and Henri Bergson’s philosophy—who is regarded as a philosophical predecessor to contemporary views of process philosophy (Meincke 2022; Seibt 2022).

To be fair, it is known that Husserl did not know of Bergson’s philosophy by 1907, the year in which he gave the lectures on inner time-consciousness that constitute the core of Husserl (1991) (see Spiegelberg 1994, p. 107). Husserl’s phenomenological analyses of subjective temporality seem to be more influenced by both Kant and James (Brough 1991). However, despite apparently his ignorance of Bergson’s works, it is known that Husserl stated in 1911 that ‘we are the true Bergsonians’ after listening to Alexandre Koyré’s report on Bergsonian philosophy (Héring 1939, 368; Spiegelberg 1994, p . 428), as well as ‘It is precisely as if I were Bergson’ when listening to Roman Ingarden’s account of Bergson’s pure duration in his doctoral dissertation (which Husserl was supervising) in 1917 (Ingarden 1968, p. 121). It is these similarities that apparently Husserl saw between his own views on subjective temporality and the philosophy of Bergson when it was presented to him by other people what we want to exploit here. It should be acknowledged, however, that despite how similar their views on temporality are (see Winkler 2006; Kelly 2010), there are numerous differences between Husserlian phenomenology and Bergsonian philosophy (see Lovasz 2021).

The critical insight to which Bergson arrives at is that time, in contrast to space, is entirely indivisible. In space, anything can be juxtaposed and divided in the sense of geometric series. For time, however, this is not the case. Time is considered to differ not in ‘degrees’ akin to space but in ‘kinds’ (i.e. not in magnitude, but in kinds of units). Changes in space are quantitative, while changes in time are qualitative. Thus, for Bergson, two temporal moments are different in kind; they are qualitatively incomparable, like comparing oranges to apples. How can, then, human experience appear coherent and continuous?

Because human experience is considered a process, Bergson resolves the conundrum by introducing ‘durée’, which is built upon the more fundamental solution to continuity in process, i.e. the interpenetration of time (Costelloe 1912; Mullarkey 2000; Bergson 2001). This is a critical step towards his idealistic concession when studying consciousness (i.e. perception) as a process. Only temporal analysis can provide insights into how consciousness comes to be. For Bergson, any moment is directly integrated with other moments, making it entirely mobile, as it were, which in turn makes every moment unique (Bergson 2007, p. 131). In other words, the reason continuity occurs is due to the integration of past and future moments.

The continuity of time as analysed by Bergson is captured by Husserl’s (2001b, §3) notions of retentional modification and protentional fulfilment. The idea is that what is protended and retained within the living present is not to be understood as a set of discrete points but rather as a continuous flow of experience. Put simply, at any given moment, we are aware of the about-to-occur via protentional intentions. Such anticipatory awareness [which is characterized by Husserl as somewhat indeterminate (Husserl 2001a, §10, 2001b, p. 46)] is fulfilled (or negated) in primal impression. That same primal impression is immediately modified retentionally, which is to say that it continually loses its experiential vivacity until it eventually sinks into an undefined past (Husserl 2001a, §35). In other words, the path from protention to retention is better understood as a ‘continuous flow’ or ‘process’ of intentional modification.

Despite Husserl’s insistence on the continuous nature of the structure of time-consciousness, he used to represent such structure by using geometric diagrams (Fig. 1). This schematic sums up his views on the temporal structure of experience while also accounting for the interpenetration Bergson talked about. Husserl does so by rendering any present moment as being constituted by the integration of the immediate past and future. Such a present moment (i.e. the living present) is experienced as a continuous flow, given the incessant process of intentional modification.

Based on the aforementioned analysis, Husserl goes on to distinguish three distinct levels of temporality and the constitution of objects in time (Husserl 1991, §34):

1. The empirical objects found in objective time
2. The immanent objects constituted in subjective temporality
3. The ‘absolute’ flow of consciousness

For Husserl, each level is constitutive of the aforementioned one. The thought is that we can only make sense of an objective time in which there are empirical objects (e.g. the objects of physics) because we experience objects as appearing immanently in the living present.

It should be acknowledged that Husserl returned to question of how objective time is constituted subjectively repeatedly during his lifetime. We focus on his early approach to the matter on Husserl (1991), which is limited to individual experience. In accordance with his own later analyses on intersubjectivity and the life-world, one should probably approach the constitution of objective time in relation to those topics (Rodemeyer 2006). This kind of approach, however, is beyond our scope in this paper.

It is in the interplay between protentional fulfilment and retentional modification that objects are constituted in subjective temporality (i.e. in the living present). Insofar as what is protended is confirmed in primal impression, a ‘synthesis of fulfilment’ occurs (Husserl 2001a, §16). In other words, what was anticipated is identified as identical to what is given in the now-phase of experience. Similarly, given the process of retentional modification, what was given in primal impression maintains its individuality but is now experienced as having-occurred until it sinks into the undetermined past. Thus, in the flow of subjective temporality, the experienced object is constituted as maintaining its identity via intentional syntheses.

The crucial step from the constitution of the objects in subjective temporality to the constitution of objective time and its objects lies in the fact that, according to Husserl, each ‘[…] now-moment is characterized above all as the new. The now that is just sinking into the past is no longer the new but that which the new has pushed aside. In this being-pushed-aside there lies an alteration’ (Husserl 1991, p. 65). What is given in primal impression is experienced as always new, which does not mean that in subjective temporality, we are dealing with discrete time chunks, but with continuous now-moments that are qualitatively distinct from one another, given the processes of protentional fulfilment and retentional modification. What was given in the just-past is now experienced as just-past via retention, which is qualitatively different from what is given in primal impression. It is because of this qualitative difference that each current now-phase creates a new fixed point in time, giving rise (i.e. constituting) that objective time conceived of as a rigid ordering of such fixed points and what is given in them. The intertwining of subjective temporality and objective time is depicted in Husserl’s diagram of time (Fig. 1).

By making use of diagrams to represent time and experience, we may accidentally spatialize it and thus treat it as a ‘thing’ instead of a continuous process. Perhaps, as Bergson and Husserl suggest, the temporal structure is best thought of as events that do not share the same unit with their predecessor or successor. To be sure, we strategically only number the events relative to a reference.

From the aforementioned analysis, we may strategically generate two phenomenological criteria for computational models:

1. Any moment in time that is experienced as ‘the present moment’ must be constituted by properties that correspond to the just-past (via retentions) and the about-to-occur (via protentions). That is, any experienced moment in time cannot consist of a discrete ‘now’ moment alone but must instead coexist with the immediate past and immediate future.
2. Any moment in time that is experienced as ‘the present moment’ consists of the intertwining of subjective temporality and objective time. That is, the present moment requires the coming together of the subjective and objective temporal structures.

## Objective time: thermal time

In contrast to subjective temporality, and outside of phenomenological analyses, objective time pertains to the temporally sequential development of worldly processes bound relatively to space (i.e. space-time). The fact that computational models behave according to these laws, as they pertain to worldly processes, is precisely the incongruence between subjective temporality and objective time that we seek to resolve. In this section, we briefly present a possible way in which objective time can be made sense of from a physical (i.e. non-phenomenological) perspective that is consistent with the active inference framework. We acknowledge, however, that one does not have to subscribe to the view presented in this section to appreciate the link between subjective temporality and objective time within the active inference framework (see ‘An Active Inference Model of Temporality’).

We emphasize two important properties of objective time, namely, its spatiality and its directionality (Eddington 2019). Spatiality simply means that time is considered with reference to space, so that it follows spatial laws, and it is thus divisible similarly to space. In contrast to continuous flows, any two moments of time can be said to be discretely separated, so that a discontinuity between them occurs. This physical conception of time allows us to separate time frames and measure them. For instance, we can talk about a time frame that lasts 23 s, where each second is disjointed from one another. More importantly, due to its worldly and spatial status, objective time can thus be understood to follow thermodynamic laws providing it with a direction. The direction stems from the laws of thermodynamic entropy (i.e. heat transfer towards cold) that the difference in entropy between two moments of objective time is either the same or increased, forming the central argument in the ‘thermal time hypothesis’ proposed by Rovelli (1993; see also Connes and Rovelli, 1994). As an extension, Jeffery *et al.* (2019) suggest that this may be a critical insight into the flow of physical systems, including biological ones. Living things have metabolisms governed by the second law of thermodynamics, which is a microscopic variable. Macroscopic variables of structure, however, are viewed as mechanisms that foster entropy via metabolism since they are entropically favoured. Their view establishes a bridge between the thermodynamic arrow of time and an information-entropic one that fits the biochemical processes necessary for human experience—quite similar to the free energy principle (Friston 2005) of which active inference is a corollary (Parr *et al.* 2022). This ties the discrete and mechanical structure of objective time and the flowing common sense of temporal development as experienced. The major challenge, however, remains to propose a structure for how a sequential structure of time is compatible with one that appears to flow. This thermodynamic and entropic view of time is, nonetheless, an important first step. For alternative views on the direction and reality of time, see Barbour *et al.* (2014) and Smolin (2013).

From this perspective, objective time simplifies the structure of time as each moment in time can be said to cause the next one, suggesting a linear causal relation (i.e. from past to future), although it may look like the other way around due to entropic relations. There are thus two structures of time apparent. One describes the experienced flow, while the other describes the sequential and mechanical passing from past to future. Distinguishing between such systems is particularly significant for the following section where we undertake a temporal analysis of active inference, through a probabilistic network diagram (Pearl 1988) of time.

## An active inference model of temporality

### A general introduction

The active inference framework is a corollary of the free energy principle (Friston 2005, 2010), which takes a Bayesian optimization attitude towards both human cognition and cortical responses (or a detailed description of the framework, see Parr *et al.* (2022)). Perhaps, the most important project for active inference is the biological validation and plausibility, which rests on the success of mapping mathematical parameters onto empirically measurable properties of the body and brain. Thus far, several complex behaviour performances and dynamics have been successfully simulated under active inference (for a review, see Da costa *et al.* 2020).

In short, active inference assumes a generative model (which comprises the physical structure of the system with its sensory and active states) to infer the most likely causes of observed outcomes in a generative process (which corresponds to processes in the world). The generative model can be summed up as the probabilistic mapping of how outcomes follow from causes that are hidden. In modelling a process, the system is minimizing its free energy (i.e. the information-theoretic difference between the estimated probability distributions that act on the system and probability distributions of the sensed states) through a cascade of Bayesian inferences and actions. This is the main task of active inference.

Active inference presupposes a separation between a given system and its environment. Within the model, such a separation is drawn via the Markov blanket formalism (Pearl 1988). In a Bayesian network, a Markov blanket represents the statistical boundary between a set of variables (dubbed internal states) and a further set of variables (dubbed external states) to which the former are conditionally independent. This means that if we know the values of the blanket states, knowing the values of the external states would not give us any new information of the internal states. Markov blankets are typically applied to the network domain of systems to identify the spatial (statistical) boundaries of, for instance, cells and neurons (Clark 2017; Kirchhoff *et al.* 2018; Hipólito *et al.* 2021).

Can a Markov blanket be applied to Husserl’s model of temporality to identify the present moment? In dealing with time, the system itself is not of a spatial kind. However, due to the directionality of objective time, we can turn the temporal model into a directed acyclic graph (DAG) that describes the conditional dependencies between past and future. This fact justifies the use of Markov blankets to statistically define the boundaries of the present moment.

### Integrated continuity and conditional dependencies

In recent developments of the active inference framework, the temporal dimension of such models has been further elaborated and discussed (de Vries and Friston 2017; Friston *et al.* 2018). In these models, the temporal aspect of active inference is summed in the ‘belief updating’ protocol, where the agent takes a step forward in time, updating its beliefs via belief propagation about external states based on internal states. At first sight, this process appears similar to other Markov decision processes where the temporal development follows a sequential order, and each state is static in the sense that it has no relation to other time points than the preceding state. However, in this and the following sub-sections, we show how active inference’s temporal development differs.

**Figure 2.**
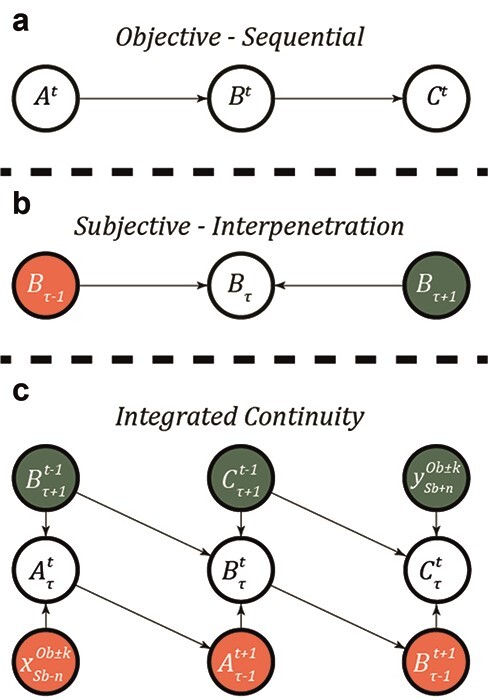
This schematic illustrates three kinds of conditional dependencies. (a) This conditional dependency illustrates the conventional sequential temporal structure in objective time. In the terminology of causal inference, this process is a convention causal path. (b) This conditional dependency illustrates an interpenetrated temporal structure as discovered in Husserl’s model of time. In the terminology of causal inference, this process can be described as a collider. In the context of causal inferences, conditional dependencies within a Bayesian network are usually taken to map onto causal processes (Peters *et al.* 2017, pp. 105–106). Thus, it may be assumed that some of the conditional dependencies described here map onto causal processes related to objective time. Notice, however, in relation to a phenomenological analysis of subjective temporality, the relationships between nodes are not causal but of ‘motivation’. From the phenomenologists’ perspective, the relationships between phenomena that they are interested in are not of the natural kind because they are subjective. Causality, in contrast, is a natural and objective process in the world. For this reason, phenomenologists often avoid talking about causality and prefer the concept of ‘motivation’: ‘One phenomenon triggers another not through some objective causality, such as the one linking together the events of nature, but rather through the sense it offers’ (Merleau-Ponty 2012, p. 51). The link between causality, motivation, and conditional dependencies may prove to be crucial for the naturalization of phenomenology and the phenomenologization of science from the perspective of computational phenomenology. As stated by Carel and Meacham, it is the ‘relation or non-relation between the orders of causation (nature) and motivation (experience) that remains the decisive issue in any exploration of the relationship between phenomenology and naturalism’ (Carel and Meacham 2013, p. 7). As we see it, in the active inference framework, conditional dependencies may serve as a bridge between motivation and causality. A full-fledged elaboration of this idea remains nevertheless beyond the scope of this paper. (c) This conditional dependency illustrates an integrated continuity taking both models into careful consideration. Note here that objective time is declared in the superscript, whereas subjective temporality is declared in the subscript: $x_{Subjective\,temporality}^{Objective\,time}$. Note also that objective time may be self-referenced, whereas human time is always related to some (conscious) event

Thus far, we have presented two temporal structures. When focusing on its physics alone, (objective) time is conceived of as following a ‘sequential order’. In contrast, when focusing on how it is originally experienced, subjective temporality follows an ‘interpenetrated order’. By diagrammatically drawing up the temporal relationship between them, an integrated version becomes apparent in the form of a Bayesian network (Fig. 2). As we merely analyse the temporal structure of the framework, we omit here the whole generative model, which can be found in thorough papers on active inference (e.g.; Friston *et al.* 2017). If we treat each node in Husserl’s model as a random variable, we get an interesting chain of relations that can be translated into a DAG. This effectively reveals the conditional dependencies between retentions and protentions in their constitution of the present.

We distinguish the following temporal orders:

- **Sequential order** is characterized by a unidirectional development in time guided by the direction of objective time where the current event depends on the prior event.
- **Interpenetrated order** is characterized by a bidirectional development in time guided by the human predictive attitude where the current event depends on both the prior and posterior events.
- **Integrated continuity** is a combination of both orders suggesting a bidirectional development in time at each event while advancing in a unidirectional manner. This effectively corresponds to the integration between subjective temporality and objective time. In other words, this integration serves as a model of how, within Husserlian phenomenology, subjective temporality is constitutively intertwined with objective time.

When modelling the temporal structures of objective time and subjective temporality in terms of conditional dependencies, a third option becomes clear, namely, the integrated continuity. This conditional dependency presents a temporal structure that includes features from both sequential and interpenetrated structures. It is essentially a temporal structure that promises the potential of integrating objective time and subjective temporality within the model. Although surprisingly reminiscent of Husserl’s diagram of time, the major difference lies in the notations. By continuing to distinguish between objective time and subjective temporality at any node, we keep track of the temporal development relative to both an objective and a subjective reference point. This is essentially the tool to which active inference is matched and analysed.

While the sequential structure appears straightforward, the interpenetrated structure may pose a problem. In the terminology of causal inference, it corresponds to a ‘collider’ (Pearl 1988). In short, when having two causes to an effect, it is necessary to assess the relation between the causes. As this is under subjective temporality, it corresponds to asking what the relation is between retention and protention. It is here that the rules of causal inference fall short as they mainly pertain to spatial relations.

The integrated continuity has an asynchronous structure with a temporal evolution at its focus. As retentions stem from past primordial impressions that in turn stem from joint previous protentions and retentions, the integrative circularity assures us a conditional link constantly integrating the past with the future (Fig. 2c). By this token, the relationship between the two causes is known. Within the model, this is a consequence of the directionality of the objective time following the flow of energy. By considering temporal development in an integrated fashion, we may begin to answer how multiple processes make up the present. How does this temporal structure map to the temporal structure of active inference?

### A temporal analysis of active inference

**Figure 3.**
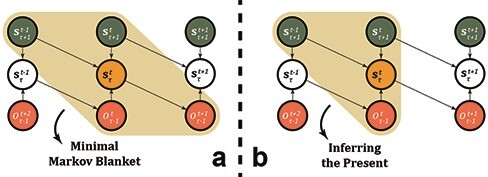
This schematic is a graphical description of the revision of the beliefs about the variables in light of new data. It described the statistical dependencies at a particular node in the integrated continuity. (a) By treating the continuity in terms of conditional dependencies, we may knit a Markov blanket to determine the system constituting the present moment to elucidate the statistical boundaries of the present moment. We effectively consider protention and primal impression as random variables, while the nodes of retention are considered observations or empirical priors in line with active inference. First, it is worth noticing that the blanket exemplifies how the present moment can be described through the integrations of the immediate past and future. Second, it may be observed that an asynchronous structure emerges. The yellow area illustrates the blanket. (b) In computing the probability of the present moment, the DAG suggests that we simply need three other states. The yellow area illustrates the nodes necessary to infer the present. Notice here that everything in retention is signified as $o_{Sb - n}^{Ob \pm k}$ as it now belongs to observed states rather than inferred states, whereas the inferred sensory states are signified as ${\bf{s}}_{Sb + n}^{Ob \pm k}$, where *Ob* refers to the objective time, *Sb* refers to the subjective temporality, and *k* and *n* refer to the number of steps in each direction

In determining the statistical boundaries of the present, we first assess the Markov blanket under the integrated continuity of time to assess the relationship between subjective temporality and objective time as well as retention and protention (Fig. 3a). This is then followed by considering the computation of the probability of the present. The blanket of the present moment merely declares that if we know the states within the blanket, then no other variable in the system can provide additional information about the present moment. Keep in mind that this only reveals the dependencies of the system when certain states are observed. To infer the present moment, however, we need to compute the probability of ${\bf{s}}_\tau ^t$ given a set of observations (Fig. 3b).

In Fig. 3, the integrated continuity has been mapped to fit both objective time and subjective temporality around a reference point (yellow node). All (green) nodes above designate the protention relative to the (white) central nodes, while all (red) nodes below designate the retention also relative to the (white) central nodes. The direction of time is from left to right in our aforementioned example.

In assessing the blanket, we observe that the blanket suggests an asynchronous structure where an imbalance between past and future states assures continuity in the present state through time. This finding suggests that even in a minimal Markov blanket, elements of the past are preserved in the present and future states in an eschewed manner that asserts a continuous flow mirroring Husserl’s analyses. This speaks to the potential of applying statistical schemes to temporal structures converted into conditional dependencies. Additionally, in assessing the computation of the present state, it appears to fit the update function of active inference in a surprisingly meaningful way. If we only consider the computation of the present moment from the integrated continuity:


(3.1) $$p\left( {{\bf{s}}_\tau ^t,{\bf{s}}_{\tau + 1}^{t - 1},{\bf{s}}_{\tau + 1}^t,o_{\tau - 1}^t} \right) = p\left( {{\bf{s}}_{\tau + 1}^{t - 1}} \right)\,p\left( {{\bf{s}}_{\tau + 1}^t} \right)\,p\left( {o_{\tau - 1}^t} \right)\,p\left( {{\bf{s}}_\tau ^t{\rm{|}}{\bf{s}}_{\tau + 1}^{t - 1},{\bf{s}}_{\tau + 1}^t,o_{\tau - 1}^t} \right)$$

(3.2) $$\ln {\bf{s}}_\tau ^t = \ln {\bf{s}}_{\tau + 1}^{t - 1} + \ln {\bf{s}}_{\tau + 1}^t + \ln o_{\tau - 1}^t$$

(3.3) $${\bf{s}}_\tau ^t = \sigma \left( {\underbrace {\ln {\bf{s}}_{\tau + 1}^{t - 1}}_{protention} + \underbrace {\ln {\bf{s}}_{\tau + 1}^t}_{\begin{array}{*{20}{c}}
{primal}\\
{impression}
\end{array}} + \underbrace {\ln o_{\tau - 1}^t}_{retention}} \right)$$

Here, $\sigma $ refers to the softmax operator. With the compatibility between Husserl’s diagram of time and the integrated continuity, the present moment can be described using a combination of both. The joint probability of the present moment, as depicted in Fig. 3, is given by Eq. (3.1). This is a conventional expression in Bayesian networks. To stay consistent with the active inference formalization, Eq. (3.1) can be rewritten in terms of natural logarithm, where multiplication is exchanged with addition. Doing so leads us to Eq. (3.2). Finally, we apply the softmax operator as it is a way to map the output of the network to a probability distribution over a set of classes. In short, the present moment can be inferred using the protention, the primal impression, and the retention relative to the present moment. As the following section will demonstrate, Eq. (3.3) corresponds in principle to the belief propagation function in active inference, however, without any action policy in consideration.

### Living inferences

When considering the belief update mechanism of active inference, it becomes clear that it appears to follow a similar temporal structure as the integrated continuity in updating the beliefs of the agent. Note that the active inference framework reduces to a simple hidden Markov model once the active bit is taken out. Our analysis thus applies in principle to all hidden Markov models that have a similar updating scheme. Consider now the dynamical process leading to the belief propagation without the action policies:


(3.4) $${{\bf{s}}_{\tau}} = \sigma \left( {{{\bf{v}}_{\tau}}} \right){\bf{\it{\,}}}$$

(3.5) $${{\boldsymbol{\dot v}}_{\tau}} = \ln {{\bf{B}}_{{\tau} - 1}}{{\bf{s}}_{{\tau} - 1}} + \ln {{\bf{B}}_{\tau}} \cdot {{\bf{s}}_{{\tau} + 1}} + \ln {\bf{A}} \cdot {o_\tau } + \ln {{\bf{s}}_{\tau}}$$

The stationary solution can be written as follows:


(3.6) $${{\bf{s}}_{\tau}} = \sigma \left( {\underbrace {\ln {{\bf{B}}_{{\tau} - 1}}{{\bf{s}}_{{\tau} - 1}}}_{\begin{array}{*{20}{c}}
{prior}\\
{\left( {forward} \right)}
\end{array}} + \underbrace {\ln {{\bf{B}}_{\tau}} \cdot {{\bf{s}}_{{\tau} + 1}}}_{\begin{array}{*{20}{c}}
{predicted}\\
{\left( {backward} \right)}
\end{array}} + \underbrace {\ln {\bf{A}} \cdot {o_\tau }}_{likely\,outcome}} \right)$$

This can further be expressed in terms of a discretized temporal system as follows, where the subscript expresses subjective temporality and the superscript expresses the objective time:


(3.7) $$\begin{aligned}& {\bf{s}}_{\tau}^{{\rm{t}} + \Delta {\rm{t}}} \approx \nonumber\\
& \sigma \left( {\ln {\bf{s}}_{\tau}^{\rm{t}} + \Delta t\left( {\underbrace {\ln {{\bf{B}}_{{\tau} - 1}}{\bf{s}}_{{\tau} - 1}^{\rm{t}}}_{\begin{array}{*{20}{c}}
{prior}\\
{\left( {forward} \right)}
\end{array}} + \underbrace {\ln {{\bf{B}}_{\tau}} \cdot {\bf{s}}_{{\tau} + 1}^{\rm{t}}}_{\begin{array}{*{20}{c}}
{predicted}\\
{\left( {backward} \right)}
\end{array}} + \underbrace {\ln {\bf{A}} \cdot {o_\tau }}_{likely\,outcome} - \ln {\bf{s}}_{\tau}^{\rm{t}}} \right)} \right)\end{aligned}$$

Equation (3.7) considers the change in the dynamic process by discretizing the temporal difference in the development. The belief about time $\tau $ at time $t + \Delta t$ depends on the protention, retention, and primal impression. It also states that the marginal posterior over hidden states can be approximated by considering (i) the empirical priors of the immediate past state, (ii) the predictions about the future state, and (iii) the likelihood of an observation. These are also the parameters of the stationary solution in Eq. (3.8). For readability, we have maintained the archetypical notations of active inference papers, i.e. bold matrices refer to conditional probabilities, italic font type represents hidden states, and bold variables represent expected states (Table 1). Now, by carefully mapping the stationary solution of the updating function to the integrated continuity, it can be more precisely rewritten as follows:

**Table 1.** List of variables and their respective descriptions.

Variable	Description
${\bf{s}}_\tau ^t = q\left( {{s_\tau }} \right)$	State belief at subjective time *τ* and objective time *t*
$o_\tau ^t$	Outcome state at subjective time *τ* and objective time *t*
$\ln {\bf{A}} = p(o_\tau ^t|s_\tau ^t)$	Likelihood matrix, which maps states to outcomes
$\ln {\bf{B}} = p\left( {s_\tau ^{t + 1}{\rm{|}}s_\tau ^t} \right)$	Transition matrix, which maps states to states
$\sigma \left( \cdot \right)$	Softmax operator, which maps values to a probability distribution, so the sum of probabilities sum to 1
${{\bf{v}}_{\tau}} = \ln {\bf{s}}_\tau ^t$	Stationary solution to the ordinary differential equation that satisfies the belief propagation scheme of active inference (for more details, see Friston *et al.* 2017)
${{\dot v}_{\tau}} = \frac{{\partial {\bf{F}}}}{{\partial {\bf{s}}_\tau ^t}}$	Dynamics of the updating scheme, which corresponds to a gradient descent on the variational free energy
**F**	Variational free energy

Variables in italic refer to hidden states, whereas bold ones refer to expectations about those states.


(3.8) $${\bf{s}}_{\tau}^{\rm{t}} = \sigma \left( {\underbrace {\ln {\bf{B}}_{{\tau} + 1}^{{\rm{t}} - 1}{\bf{s}}_{{\tau} + 1}^{{\rm{t}} - 1}}_{\begin{array}{*{20}{c}}
{prior}\\
{protention}
\end{array}} + \underbrace {\ln {\bf{B}}_{\tau}^{\rm{t}} \cdot {\bf{s}}_{{\tau} + 1}^{\rm{t}}}_{\begin{array}{*{20}{c}}
{predicted}\\
{primal\,impression}
\end{array}} + \underbrace {\ln {\bf{A}} \cdot o_{\tau - 1}^{\rm{t}}}_{\begin{array}{*{20}{c}}
{likely}\\
{retention}
\end{array}}} \right)$$

This equation allows for a proper analysis of the temporal development in the belief propagation equation of the current form of active inference. First, it can be observed that active inference conforms to the phenomenological criteria by (i) implicitly integrating both dimensionalities of time (i.e. past and future) into the model, paving the way for a promising approach to modelling human experience and (ii) intertwining objective time with subjective temporality. Taken together, this careful mapping between the update function and the integrated continuity demonstrates meaningful phenomenological labelling of parameters making active inference particularly interesting for computational phenomenology. Notice here that the mapping has only relied on the temporal notations from both the original stationary belief updating equation (3.6) and the corresponding function under integrated continuity (3.3). Surprisingly, we discover that both models appeal to the same nodes in the temporal structure, although in different ways. This compliance makes the mapping procedure rather straightforward. The ‘prior (forward)’ in Eq. (3.6) maps to the ‘protention’ in Eq. (3.3), essentially describing the ‘prior protention’ in Eq. (3.8). Due to the integrative view we propose, this part of the equation can be understood as referring to the protention (i.e. the immediate anticipation) of the immediate past, i.e. the prior protention. The ‘predicted (backward)’ maps to the ‘primal impression’ in Eq. (3.3), which can be described as the ‘predicted primal impression’ in Eq. (3.8). Based on the integration between currently incoming sensory states and the expected state, the primal impression becomes, according to our view, a predicted primal impression. Finally, the ‘likely outcome’ in Eq. (3.6) maps to the ‘retention’ in Eq. (3.3) that can be translated into ‘likely retention’ in Eq. (3.8). Similar to how protention was brought backward, the retention is here brought forward in time, linked with the likelihood and the hidden state. This gives rise to an integrated structure of time where the past depends on my prior predictions and the future of my likely retention, which speaks to the phenomenological understanding of experience. Just as in the Husserlian analysis that retention, primal impression, and protention jointly make up the ‘living’ present, we refer to the set of inferences presented here as ‘living inferences’ since jointly they serve to describe within an active inference model the phenomenological processes that give rise to the living present. Additionally, the notion of living inferences conveys the Janus-faced/unifying nature of our proposal: it is both phenomenological (i.e. lived) and computational (i.e. inferential).

One way the intertwining of objective time and subjective temporality (3.7 and 3.8) could be read is as a misunderstanding of the relationship between the two dimensionalities of time. Similar to Husserl’s formulation of the temporal problem, active inference may have assumed a synchronous temporal change between the agent and the generative process, which is problematic in generating the continuity of experience. However, the structure of the belief updating function has been demonstrated to account for both dimensionalities of time. The correct way to read this intertwining is thus as an implicit integration of both dimensionalities of time into a holistic and diachronic view of belief updating. This kind of updating includes both the agent’s temporality as well as the generative process as explicitly stated by Friston *et al.* (2017). In short, the integrated continuity allows mapping the objective time to subjective temporality, which in turn provides deeper insights into the modelled experience.

## Possible applications

In this final section, we briefly review possible applications of our analysis of the model (and, more broadly, computational phenomenology) within consciousness studies.

Under various altered states of consciousness (e.g. dreaming, drug intake, schizophrenia, and many more), the sense of the (living) present can be distorted. From our perspective, the distorted sense of the living present can be modelled as the malfunctioning of the integrated continuity. In other words, the Markov blanket of the present may be changed in some way that can be inferred.

In several psychiatric studies, it has been emphasized how the sense of an integrated temporal continuity is disturbed (Fuchs 2007; Sass and Pienkos 2013; Stanghellini *et al.* 2016). For instance, in disorders like schizophrenia, the subjective experience of temporal continuity is reported as incoherent and discontinuous. A patient with a diagnosis of schizophrenia reports as follows: ‘While watching TV it becomes even stranger. Though I can see every scene, I don’t understand the plot. Every scene jumps to the next, there is no connection. The course of time is strange, too. Time splits up and doesn’t run forward anymore. There arise uncountable disparate now, now, now, all crazy and without rule or order’ (Fuchs 2007, p. 233). For some patients with the same diagnosis, these disturbances further extend into the meaning of sentences whose natural extension in time demands an integrated continuity for any meaningful outcome: ‘I can concentrate quite well in what people are saying if they talk simply. It’s when they go into long sentences that I lose the meanings. It just becomes a lot of words that I would need to string together to make sense’ (Fuchs 2013, p. 85).

We suggest that our computational model can also be applied to the question of temporal stability in perception, known as ‘serial dependence’, to better understand how the present is directly related to retentional capacities in the visual cortex (Cicchini *et al.* 2018). In the context of interrelationships between sensory information and time, sensory systems exploit spatial redundancies by shifting their responses to match stimulation statistics. Serial dependence of perception has provided direct evidence of how a system integrates prior information into the perception of the current one. The dependence allows biasing our perception by retaining prior information in the visual cortex (Cicchini *et al.* 2014, 2018; Fischer and Whitney 2014; Glasauer and Shi 2022). It has been demonstrated to affect the visual judgement of orientation (Fischer and Whitney 2014; Cicchini *et al.* 2017; Fritsche *et al.* 2017), body size (Alexi *et al.* 2018), eye gaze (Alais *et al.* 2018), visual variance (Suárez-Pinilla *et al.* 2018) and confidence (Rahnev *et al.* 2015). One important limitation is that the serial dependence has only been tested in the visual modality and is thus not necessarily a general principle. If our account is correct, which assumes the same underlying temporal architecture in sensory systems, then it predicts that the serial dependence is also observable throughout other sensory modalities.

## Concluding remarks

In this paper, we evaluated the structure of subjective temporality analysed by Husserl and derived a set of criteria that a computational model must adhere to in order to be phenomenologically meaningful. From our perspective, a properly phenomenologized model of time-consciousness must comply with the criteria presented here. We then analysed and compared two types of temporal structures—a sequential (objective time) and an interpenetrated (subjective temporality)—and suggested an alternative version, i.e. integrated continuity. By relying on this alternative structure, we demonstrated that the belief updating equation of active inference adheres to the set of phenomenological principles licensing active inference to model phenomenological subjective values on a temporal note. In a word, the integrated continuity in the belief update equation of active inference suggests that such an integration embodies the living present, however, as a form of living inferences. That is, as time continues to unfold, the continuous integrations of retention and protention become living inferences. The close relationship between past and future within the present has been observed to be of high importance in other computational and empirical studies (Jazayeri and Shadlen 2010; Shi *et al.* 2013; Fischer and Whitney 2014; Djebbara *et al.* 2022; Glasauer and Shi 2022).

We speculate whether the fact that inferences in this context are always directed towards the future corresponds to what Husserl once stated: ‘Generally, and without further ado, we see that every intention whatsoever is anticipatory, and this feature is due precisely to the striving that, as such, is directed towards something that can only first be achieved through a realization’ (Husserl 2001a, p. 130). Based on the aforementioned analysis, we recognize that the temporal structure of active inference complies with the temporal structure suggested by Husserl in his phenomenological analysis of human temporality. In contrast to several computational models, active inference is particularly unique in its principled predictive and integrated architecture of inferences. Furthermore, the analysis also provided temporal grounds for conceiving active inference as a temporally asynchronous process. It is essentially based on this temporal architecture that we render it compatible with human phenomenology. Perhaps, as noted by Kent and Wittmann (2021), the temporal domain of theories of consciousness lacks a central component, namely, the temporal dimension. As we see it, active inference is developed as a process theory similar to evolution as it describes the processual changes necessary to develop the skills that we have obtained today and that we will obtain tomorrow.

An important consequence of this analysis is the justification of the mapping between estimated parameters and human experience to further assess the experience of, for instance, patients with psychiatric disorders. If the mapping between the parameters of active inference and neuronal structures proves to hold (Parr and Friston 2017a, 2017b, 2018a, 2018b), then not only we may begin to understand the important changes and differences of psychiatric disorders, but we may also finally begin to understand them from within. For instance, patients with the schizophrenic disorder report an impaired temporal grip (Giersch *et al.* 2013, 2015; Allé *et al.* 2016) and a disturbed sense of self (Lysaker and Lysaker 2001; Henriksen and Parnas 2012). Under the ‘disconnection hypothesis’ (Friston and Frith 1995), we may understand how these impairments of self-continuity and the integration of information are experienced. In general, it allows for assessing, monitoring, and understanding the subjective values attributed to, and associated with, certain impaired cognitive skills. Given the proposal put forth in this paper, this connection between the experience of temporal continuity and the phenomenology of certain psychiatric disorders opens an interesting and important direction for future research and application of computational phenomenology.

A further direction for future research has to do with the broader project of the naturalization of phenomenology. As we see it, the future of computational phenomenology rests on the success of two mappings: (i) mapping the reported subjective experiences and their phenomenological analyses and the physiological properties of the body and brain onto the parameters in active inference and (ii) mapping the phenomenological subjectivity from the DAG structure imposed by active inference to empirical experiments. It is in this sense that computational phenomenology submits to a change in its attitude towards phenomenology and natural science; they are considered co-constraining and both are subject to adjustments according to empirical evidence. For this reason, this paper is a phenomenological way of reading off the dynamics of active inference, insofar as the input states refer to meaningful sensory states and the actions to bodily actions.

## Data Availability

The authors confirm that there was no data involved in our work and thus no data to make available.
